# Efficacy and safety of hypofractionated radiotherapy combined with temozolomide in patients with newly diagnosed glioblastoma with poor prognosis: a single-center retrospective analysis

**DOI:** 10.3389/fonc.2026.1782296

**Published:** 2026-05-13

**Authors:** Fangmeng Fu, Li Ma, Fengming Lan, Peng Chen, Teng Zou, Ling Lei, Renba Liang, Manyi Zhu, Jianghu Zhang

**Affiliations:** 1 National Cancer Center/National Clinical Research Center for Cancer/Cancer Hospital & Shenzhen Hospital, Chinese Academy of Medical Sciences and Peking Union Medical College, Shenzhen, China; 2 National Cancer Center/National Clinical Research Center for Cancer/Cancer Hospital, Chinese Academy of Medical Sciences and Peking Union Medical College, Beijing, China

**Keywords:** efficacy, glioblastoma, hypofractionated radiotherapy, poor prognosis, temozolomide

## Abstract

**Background:**

The optimal management for newly diagnosed glioblastoma (GBM) patients with poor prognostic features, such as RPA class IV-VI or rapid early progression, remains debated. This study evaluated the efficacy and safety of postoperative hypofractionated radiotherapy (HFRT) with concurrent temozolomide in this population.

**Methods:**

Single-institution retrospective analysis (Jan 2021-Aug 2025) included patients with histologically confirmed GBM and RPA class IV-VI or rapid early progression who completed postoperative HFRT (≥3 Gy/fraction) with concurrent temozolomide.

**Results:**

Among 18 eligible patients, most had unfavorable characteristics (subtotal resection: 66.7%, biopsy-only: 33.3%). With a median follow-up of 22.3 months, the median progression-free survival was 8.4 months and median overall survival was 17.7 months. The disease control rate was 77.8%. Recurrence patterns were in-field (46.2%), marginal (38.5%), and out-of-field (15.4%). Acute grade ≥3 toxicities occurred in 16.7% of patients, all managed conservatively. No radiation necrosis was observed. Corticosteroid dependence occurred in 44.4% of patients but was manageable.

**Conclusion:**

In this exploratory single-center retrospective study, individually tailored HFRT with concurrent temozolomide demonstrated promising survival outcomes and controllable toxicity in poor-prognosis GBM patients unsuitable for standard therapy. The recurrence pattern observed in this small sample cohort suggests a potential need for optimized target volume delineation for HFRT in this population. HFRT represents a potential viable therapeutic alternative in this challenging population, warranting further large-sample prospective validation.

## Background

Glioblastoma multiforme (GBM) is the most common primary malignant neoplasm with poor prognosis in the adult central nervous system (1–3). The current standard of care for newly diagnosed GBM is based on a multi-modal approach involving maximal safe surgical resection, followed by postoperative radiotherapy in combination with concurrent and adjuvant temozolomide (TMZ) chemotherapy, with or without the addition of electric fields. Despite all efforts, the outcomes for GBM patients remain poor with the median overall survival (OS) of 14–21 months (4). Especially for those patients with poor prognosis (recursive partitioning analysis classes IV-VI and early rapid progression), the survival outcome is even worse (5–9). Limited researches have focused on these poor-prognosis patients and the best treatment needs further exploring (7, 10).

Comprehensive pathological and radiological analyses have extensively demonstrated that local recurrence is the major reason for initial treatment failure, accounting for 78%-90% of cases (11, 12). Advances in radiation oncology and radiobiological research have yielded growing evidence that hypofractionated radiation therapy (HFRT) regimens deliver a higher per-fraction dose while enabling shortened treatment schedules. These regimens may fundamentally alter tumor kinetic dynamics. Specifically, HFRT has the potential to inhibit accelerated tumor repopulation during interfraction intervals, thereby synergistically enhancing radiation-induced tumor cytotoxicity while maintaining normal tissue tolerance. Moreover, the simultaneous integrated boost (SIB) strategy in HFRT enables dose escalation to the gross tumor volume (GTV) while sparing surrounding normal brain tissue, which not only further suppresses accelerated tumor repopulation by shortening the overall treatment course and increasing per-fraction dose, but also modulates the hypoxic microenvironment of high-grade gliomas—high fractional doses can enhance the radiosensitivity of hypoxic tumor cells by inducing more severe DNA double-strand breaks, and inhibit the activation of hypoxia-inducible factor (HIF) pathways that mediate tumor angiogenesis and invasion, thus achieving a more potent anti-tumor effect.

This dual - mechanism action could significantly improve the therapeutic ratio in cancer management (13, 14). Based on the strong evidence from multiple prospective randomized trials, the National Comprehensive Cancer Network guideline recommends HFRT as a standard treatment option for elderly patients or those with poor performance status (15). Meanwhile, emerging clinical investigations are expanding the application scope of HFRT, exploring its potential use in the management of newly diagnosed GBM through both preoperative neoadjuvant and postoperative adjuvant regimens, as well as in the setting of difficult-to-treat patient populations (16).

In this study, we aim to preliminarily explore the efficacy and failure pattern of HFRT with concurrent TMZ in this specific poor-prognosis population, and to provide preliminary clinical evidence for subsequent prospective research, rather than direct guidance for routine clinical practice.

## Methods

### Patients

This retrospective study analyzed patients with glioblastomas who underwent radiotherapy at National Cancer Center/National Clinical Research Center for Cancer/Cancer Hospital & Shenzhen Hospital from January 2021 to June 2024. Inclusion criteria comprised: (1) histopathologically confirmed GBM with wild-type isocitrate dehydrogenase (IDH) 1 according to the 2021 WHO Classification of Tumours of the Central Nervous System; (2) evidence of postoperative residual or rapid progression or European Organization for Research and Treatment of Cancer (EORTC) Radiation Therapy Oncology Group (RTOG) Pretreatment Prognostic Assessment (RPA) classification, RPA class (17) IV-VI(IV: age < 50 years, KPS < 90; age ≥ 50 years, KPS ≥ 70, total or subtotal resection, good neurological function; V: age ≥ 50 years, KPS < 70, stereotactic biopsy, GBM, neurological function that inhibits the ability to work; VI: age ≥ 50 years, KPS < 70, abnormal mental status); Surgical resection extent was evaluated primarily via contrast-enhanced MRI obtained 27–72 hours postoperatively, with residual lesions on T1-weighted enhanced images serving as the core assessment index; the baseline residual tumor volume was further quantified by semi-automated volumetric analysis. The specific definitions are as follows: ① Gross Total Resection (GTR): No visible enhanced tumor residue on postoperative contrast-enhanced MRI (no abnormal enhanced foci on T1-weighted enhanced phase); ② Subtotal Resection (STR): ≥90% of the tumor volume was resected, but residual enhanced lesions were still present; ③ Partial Resection (PR): <90% of the tumor volume was resected; ④ Biopsy: Only tumor tissue was obtained for pathological diagnosis without intentional tumor resection. (3) completion of postoperative hypofractionated radiotherapy (per-fraction dose ≥3Gy) and concurrent TMZ. Exclusion criteria were: (1) prior central nervous system surgery or radiotherapy; (2) concurrent life-threatening comorbidities (e.g. progressive cerebral infarction, acute myocardial infarction); (3) coexistence of other malignant neoplasms; (4) contraindications to MRI examination; (5) incomplete clinical documentation. All treatment plans were reviewed and validated by a multidisciplinary tumor board to ensure protocol compliance.

### Treatment

All patients underwent immobilization using a customized thermoplastic head mask system for simulation. Contrast-enhanced computed tomography (CT) scans were acquired in the treatment position with slice thicknesses of 1.25/2.5 mm. Image fusion was performed between simulation CT and diagnostic T1-weighted contrast-enhanced MRI sequences obtained within 7 days prior to simulation. The gross tumor volume (GTV) was delineated as the union of contrast-enhancing lesions identified on both CT and MRI. Clinical target volume (CTV) expansion was individually optimized (0–2 cm margin) based on neuroanatomical boundaries and preoperative tumor infiltration patterns. A 3-mm isotropic planning target volume (PTV) margin was applied to account for inter-fraction setup variability. Radiation dose prescriptions were tailored through comprehensive evaluation of tumor volume, proximity to critical organs-at-risk (brainstem, optic chiasm, hippocampi), and patient performance status. Advanced inverse planning techniques were employed using either Tomotherapy (TOMO) or Volumetric Modulated Arc Therapy (VMAT) platforms, with dose calculations performed using a collapsed cone convolution algorithm. The prescription dose was normalized to the 95% isodose line encompassing the PTV, adhering to ICRU-83 reporting standards. The dose thresholds of HFRT in this study were determined based on three core foundations: (1) the definition of HFRT for central nervous system tumors in the NCCN Clinical Practice Guidelines for Central Nervous System Cancers, which recommends a per-fraction dose of ≥3 Gy as the basic dose threshold for hypofractionated radiotherapy for GBM; (2) the applicable ranges of per-fraction dose (3–4.5 Gy) and total dose (45–70 Gy) were determined based on the results of previous prospective and retrospective studies on hypofractionated radiotherapy for poor-prognosis GBM. we defined that the biologically effective dose (BED) was calculated using the linear-quadratic (LQ) model with α/β=10, and the target BED of 80 Gy was set based on the conclusion from previous studies that BED ≥70 Gy improves local control in GBM patients.(3) minor individualized adjustments were made according to patients’ tumor volume, location, proximity to organs-at-risk (OARs) and performance status; meanwhile, the SIB dose regimen was referenced from the study by Gregucci et al. (10), with slight modifications made based on the tumor’s distance from critical OARs.

During HFRT, concomitant TMZ was administered at a dose of 75 mg/m² of body surface area daily until the final day of HFRT. Where feasible, adjuvant TMZ was initiated 4 weeks after the completion of radiation therapy, with a dosage of 150–200 mg/m²/day for 5 days every 28 days.

### Follow-up and toxicities evaluation

Patients underwent initial follow-up evaluation one month post-radiotherapy completion, with subsequent assessments conducted at 2–3 month intervals. Symptomatic progression triggered immediate clinical and neuroimaging reevaluation. Standard follow-up evaluations included comprehensive medical history review, detailed neurological examination, and full neuroimaging protocol utilizing multiparametric magnetic resonance imaging (MRI) with T2/FLAIR, diffusion-weighted imaging (DWI), dynamic susceptibility contrast (DSC) perfusion, and magnetic resonance spectroscopy (MRS) sequences to differentiate true progression from pseudoprogression (PsP) and infiltrative tumor components.

For patients with suspected progression within the first 12 weeks after HFRT, a confirmatory MRI was performed at 4 weeks after initial suspicion, and final progression status was confirmed at 12 weeks post-radiotherapy unless the lesion was located outside the radiation field. Imaging findings were systematically reviewed through multidisciplinary tumor board consensus. Therapeutic response was classified according to RANO response criteria.

Standardized judgment criteria for recurrence patterns are defined based on the overlapping ratio of recurrent lesion volume and the 100% isodose line of the PTV. The specific criteria are as follows: in-field recurrence is defined as more than 80% of the recurrent lesion volume located within the 100% isodose line; marginal recurrence is defined as 20%-80% of the recurrent lesion volume located within the 100% isodose line; distant recurrence is defined as less than 20% of the recurrent lesion volume located within the 100% isodose line. Integrated multimodal functional imaging (PWI, MRS) and longitudinal follow-up were used to distinguish tumor recurrence from radiation necrosis, without reliance on surgical re-resection histopathology. All judgments of recurrence patterns were uniformly completed by the multidisciplinary tumor board consisting of radiation oncologists, neurosurgeons and radiologists in accordance with the above criteria, which ensured the objectivity, consistency and accuracy of the recurrence pattern classification results. Treatment-related toxicities were graded prospectively using National Cancer Institute Common Terminology Criteria for Adverse Events (CTCAE) version 4.0.

### Statistical analysis

PFS/OS were analyzed using the Kaplan-Meier method. All data analysis were performed using SPSS 27.0 software (IBM, Chicago, IL, USA).

## Results

### Patient characteristics

From January 2021 to June 2024, a total of 18 eligible patients (50% male, 50% female) were enrolled in this analysis. The median age of patients was 52 years (range 29-75) and a median Karnofsky Performance Status (KPS) score of 70. Surgical interventions included subtotal resection in 2 cases (11.1%), partial resection in 10 cases (55.6%), and biopsy-only procedures in 6 cases (33.3%). Notably, three surgical cases (2 subtotal resections and 1 partial resection) demonstrated rapid early postoperative progression. No patient underwent gross total resection (GTR), which was attributed to tumor location in eloquent brain regions (motor cortex, language area, basal ganglia, n=13), multifocal disease (n=1), or adjacency to the brainstem (n=4), making GTR anatomically unfeasible and surgically unsafe. MGMT methylation status was available for all patients, with 5 cases (27.8%) showing MGMT hypermethylation and 13 cases (72.2%) showing MGMT non-hypermethylation, which was consistent with the low MGMT hypermethylation rate in the poor-prognosis GBM population reported in previous studies.The median interval between surgical intervention and radiotherapy initiation was 26 days (range 14–42 days). According to RPA classification, 11(61.1%) patients had Grade IV, 2 (11.1%) patients had Grade V and 5 (27.8%) patients had Grade VI. Comprehensive demographic and clinical characteristics of the study cohort are summarized in **Table 1**.

**Table 1:** Summary of patient demographics.

Characteristics	N (%)
Number of patients	18
Median age	52 (29-75)
Males	9 (50.0%)
Females	9 (50.0%)
KPS	70 (40-80)
Location	
Frontal	6 (33.3%)
Temporal	5 (27.8%)
Parietal	1 (5.6%)
Occipital	2 (11.1%)
Central	4 (22.2%)
Extent of first surgical resection	
Subtotal resection	2 (11.1%)
partial resection	10 (55.6%)
Biopsy	6 (33.3%)
Median baseline residual tumor volume (cm³)	28.6 (8.2–61.5)
MGMT hypermethylation	
YES	5 (27.8%)
NO	13 (72.2%)
Baseline MMSE	
30	2 (11.1%)
27-29	5 (27.75%)
<26	11 (61.1%)
EORTC RPA class	
IV	11 (61.1%)
V	2 (11.1%)
VI	5 (27.8%)

KPS, Karnofsky Performance Status; KPS, Karnofsky Performance Status; MMSE, Mini-Mental Examination; EORTC, Research and Treatment of Cancer; RPA, Pretreatment Prognostic Assessment.

### Treatment

The PTV exhibited a mean volume of 308.32 cm³ (range: 85.9-588.3 cm³), with prescribed doses averaging 62 Gy (45–70 Gy). The median single-fraction dose was 3.5 Gy(3-4.5Gy), yielding a mean biologically effective dose (BED) of 80 Gy (58.5-97.9 Gy) calculated using the linear-quadratic model with α/β=10. Target volume delineation strategies varied, with 14 patients (77.8%) receiving a 2-cm isotropic expansion from GTV to CTV, while the remainder underwent focal irradiation of residual lesions owing to either an extensive involvement or poor performance status.

Fractionation regimens predominantly employed conventional schedules, with 15–22 fractions administered to most patients. The predominant fractionation scheme (n=11, 61.1%) consisted of simultaneous integrated boost irradiation: 70 Gy in 20 fractions (3.5 Gy/fraction) to the GTV with concurrent 50 Gy in 20 fractions (2.5 Gy/fraction) to the CTV encompassing the 2-cm margin.

All patients received concurrent chemoradiotherapy with TMZ(75 mg/m²) during radiotherapy, followed by adjuvant TMZ per the Stupp protocol. No patients received tumor-treating fields or molecular targeted agents during the during the first-line treatment (**Table 2**).

**Table 2:** Treatment related characteristics.

Treatment related characteristics	N (%)
RT	
Mean PTV in cc (range)	308.32 (85.9-588.3)
Mean dose (range)	62 (45-70)
Median dose per fraction (range)	3.5 (3-4.5)
Mean BED	80 (58.5-97.9)
≥2cm CTV margin	
YES	12 (66.7%)
NO	6 (33.3%)
Concomitant steroids	
Yes	2 (11.1%)
NO	16 (88.9%)
Complications	
Acute Neurological Side Effects	1
Acute non-Neurological Side Effects	2
Corticosteroid Dependency	6
Radiation Necrosis	0
pseudoprogression	
Yes	2
No	16
Site of failure at salvage re-irradiation	
In-field	6
Marginal	5
Out of field	2

RT, radiotherapy; CTV, Clinical target volume; PTV, planning target volume; BED, biologically effective dose.

### Survival outcome

**Figure 1:**
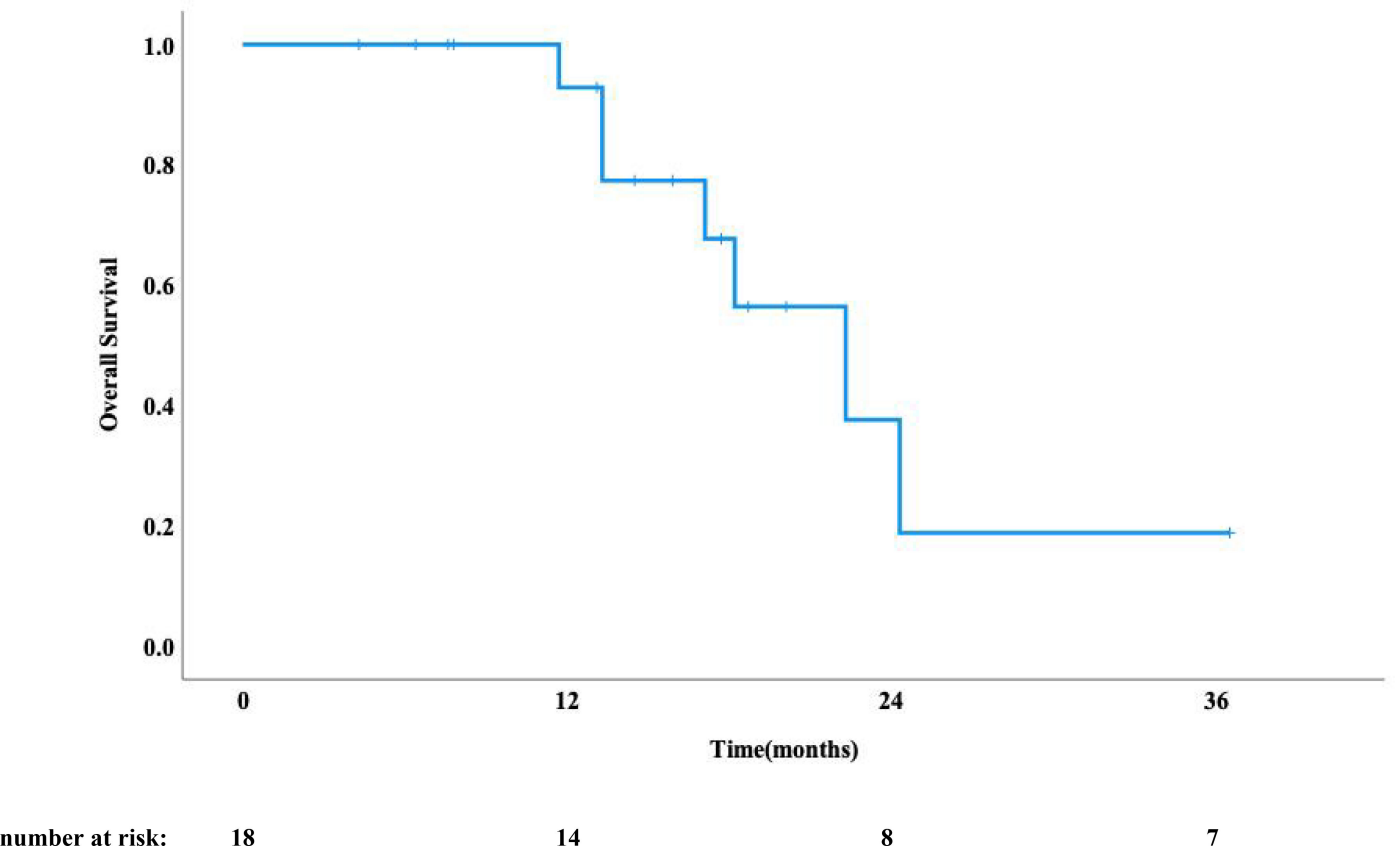
Overall survival curve.

**Figure 2:**
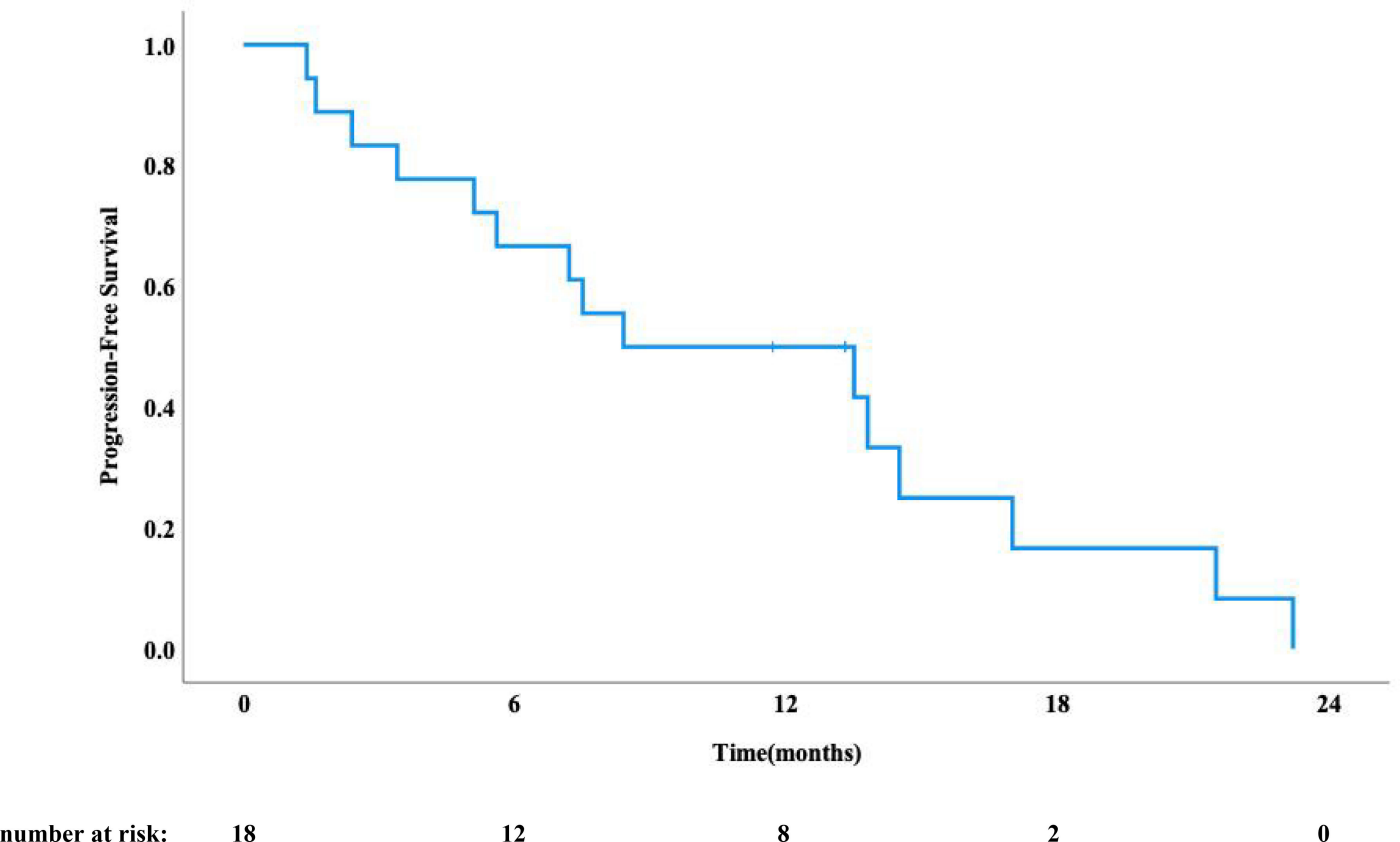
Progression free survival curve.

As of August 1, 2025, a total of 11 patients had died, while 7 remained alive. The median follow-up time was 22.3 months. The median OS was 17.7 months (95% CI: 13.7-21.7 months) (**Figure 1**). Disease progression was observed in 13 patients, with a median PFS of 8.4 months (95% CI: 5.8-10.9 months) (**Figure 2**). Analysis of recurrence patterns revealed 6 in-field recurrences (46.2% of progressed cases), 5 marginal recurrences (38.5%), and 2 out-of-field recurrences (15.4%). The median PFS durations stratified by recurrence type were 5.1 months (in-field), 9.0 months (marginal), and 2.9 months (out-of-field), respectively.

MRI-based radiographic assessment demonstrated PR in 8 patients (44.4%), SD in 6 patients (33.3%), and PD in 4 patients (22.2%). This yielded an ORR of 44.4% and a DCR of 77.8% (PR+SD) (**Table 3**). All radiological responses were assessed in strict accordance with RANO criteria, with stable or decreased corticosteroid doses in all PR and SD patients. Pseudoprogression was identified in 2 cases (11.1% of the cohort). Notably, one patient exhibited an extended PFS of 13.1 months, while another remained progression-free at the end of the follow-up period.

**Table 3:** Treatment response.

Local response	N (%)	Corticosteroid dose status at evaluation	Median KPS at evaluation
CR	0	–	–
PR	8(44.4%)	1 decreased dose/7 no corticosteroid	70
SD	6(33.3%)	2 decreased dose/1 stable dose	70
PD	4(22.2%)	2 stable dose/2 escalated dose	60
ORR	44.4%	–	–
DCR	77.8%	–	–

CR, Complete response; PR, Partial response; SD, Stable disease; PD, Progressive disease; ORR, Objective response rate; DCR, disease control rate.

### Toxicity

One patient (5.6%) developed grade 3 acute cerebral edema according to CTCAE v5.0 criteria. This case involved a multifocal tumor with histopathological confirmation via biopsy only. Severe cephalgia manifested 48 hours following radiotherapy initiation, necessitating treatment suspension. Prompt intervention with mannitol and dexamethasone achieved symptomatic resolution, enabling subsequent completion of the prescribed radiotherapy course without further complications. Two elderly patients (aged ≥60 years, 11.1%) exhibited grade 3 neutropenia during the latter phase of concurrent temozolomide-radiotherapy. Both cases were successfully managed with recombinant human granulocyte colony-stimulating factor (G-CSF), with complete hematological recovery prior to adjuvant chemotherapy initiation. Treatment compliance remained uncompromised during subsequent maintenance phases. Eight patients (44.4%) developed corticosteroid-dependent intracranial edema.

No one experienced grade 4–5 toxicity. Notably, radiographic surveillance revealed no cases of radiation necrosis.

## Discussion

This retrospective study analyzed the efficacy and safety of postoperative HFRT in 18 poor-prognosis, newly diagnosed GBM patients. The results demonstrate that in real-world clinical practice, HFRT exhibits manageable toxicities and confers a measurable disease control potential for poor prognosis patients. We also documented the recurrence patterns associated with this treatment modality.

For newly diagnosed GBM, the standard treatment was postoperative radiotherapy with concurrent and adjuvant TMZ according to Stupp protocol according with the median PFS of 6.9 months (4). However, for patients classified as poor prognosis under the RPA system, standard treatments yield limited benefits. Pedretti et al. (7) demonstrated that RPA class V-VI patients who treated with HFRT (30 Gy in 6 fractions) had a prolonged PFS of 7 months compared to TMZ monotherapy. Furthermore, Gregucci et al. (10) reported a median PFS of 8.4 months and OS of 13 months in poor-prognosis patients treated with HFRT incorporating a SIB plus TMZ, outcomes closely matching our results. Our study demonstrated a median PFS of 8.4 months and a median OS of 17.7 months in patients with RPA class IV-VI who received HFRT with concurrent TMZ. Several factors may contribute to this promising results. First, patients with RPA class IV-VI may experience tumor progression during long course radiotherapy. HFRT employs an elevated fractional dose (median 3.5 Gy) and abbreviated treatment course (median 20 fractions) with higher BED, which may mitigate accelerated tumor repopulation during radiotherapy intervals (13, 14). Second, All patients completed the regimen without discontinuation. Previous studies have demonstrated interruptions and prolonged treatment adversely affect outcome in radiotherapy. Third, 61.1% of patients received HFRT with SIB (70 Gy in 20 fractions to the GTV). The higher mean biological effective dose (BED: 80 Gy) delivered in our cohort may underlie the favorable disease control rate (DCR: 77.8%), as escalated tumor bed dosing likely enhances local control—a principle further supported by Gregucci’s observation (10). Fourth, the incorporation of TMZ likely contributed to the extended median PFS observed in our cohort. Preclinical and clinical trials have confirmed the synergistic effect of chemotherapy and HFRT. For patients with poor-prognosis GBM and a KPS ≥70, the addition of concurrent TMZ may yield additional therapeutic benefits. The large-scale trial RTOG 9006 failed to demonstrate a benefit of HFRT. In contrast to the more heterogeneous and unselected populations comprising GBM, anaplastic astrocytoma, and anaplastic oligodendroglioma enrolled in RTOG 9006, our study cohort harbored more aggressive tumors that may derive greater advantage from dose escalation. Furthermore, the high treatment compliance—wherein all patients completed the scheduled HFRT and TMZ chemotherapy without interruption—together with the aforementioned tumor characteristics, may represent critical contributors to the improved survival outcomes, beyond dose escalation and the SIB strategy. It should be noted that the exceptionally long OS in our study can be affected by potential selection bias due to the small sample size and single-center retrospective design.

Recurrence analysis revealed that marginal (38.5%) and out-of-field recurrences (15.4%) collectively constituted the majority (53.9%) of cases. This distribution contrasts with the locally dominant recurrence pattern (78-90%) documented in classical literature (11, 12). This discrepancy may arise from two factors: Firstly, individualized target volume delineation: While 66.7% of patients received a 2-cm expansion from the GTV to the CTV based on preoperative imaging and neuroanatomical boundaries, a subset (33.3%) underwent focal irradiation limited to residual lesions. This approach risks inadequate coverage of microscopic invasive foci. Secondly, The high rate of marginal recurrence strongly suggests that the 2-cm CTV expansion from GTV may be inadequate for infiltrative GBM, which is characterized by extensive microscopic tumor cell infiltration beyond the contrast-enhancing lesions on conventional MRI. Although individualized focal irradiation was used for patients with extensive tumor involvement or poor performance status to reduce normal tissue toxicity, this strategy inevitably leads to insufficient coverage of microscopic invasive foci, which is a major radiobiological risk for marginal recurrence. High fractional radiation doses may enhance local tumor control but potentially lack efficacy against distant invasive clones (18). Future studies should integrate diffusion tensor imaging (DTI) or amino acid PET to optimize individualized CTV boundaries and investigate HFRT combined with agents targeting invasive pathways (e.g. anti-VEGF therapies).

Toxicity profiles across studies further support HFRT’s safety. Pedretti et al. (7) reported predominantly mild adverse events (e.g., grade I-II headache in 77% of radiotherapy patients), while Gregucci et al. observed no grade ≥2 neurological toxicities and only three instances of grade 3–4 hematological toxicity. In our cohort, acute grade 3 toxicities occurred in 16.7% of patients (cerebral edema and neutropenia), all managed conservatively without radiation necrosis. This low incidence of severe toxicity corroborates HFRT as a well-tolerated option for frail or poor-performance-status patients, aligning with guideline recommendations for elderly/high-risk populations.

Notably, 44.4% of our patients developed corticosteroid dependence—a phenomenon potentially associated with larger planning target volumes (mean PTV: 308.32 cm³). This underscores the necessity for long-term management of radiation-induced edema, potentially incorporating adjunctive bevacizumab. Collectively, these findings suggest that while HFRT does not increase conventional radiation injuries (e.g., necrosis), its chronic effects on blood-brain barrier integrity—particularly in elderly patients or those with extensive target volumes—warrant careful clinical consideration.

MGMT methylation status is a well-recognized core prognostic and predictive biomarker for GBM, which is closely associated with the therapeutic efficacy of TMZ-based chemoradiotherapy. MGMT hypermethylation can significantly enhance the sensitivity of GBM cells to TMZ by inhibiting DNA repair, thus conferring superior survival benefits for patients receiving TMZ combined with radiotherapy. In our study cohort, the proportion of MGMT hypermethylation was only 27.8%, which was much lower than that in the general GBM population and consistent with the clinical characteristics of poor-prognosis GBM patients (RPA class IV-VI) reported in previous studies. Despite the low rate of MGMT hypermethylation, our cohort still achieved promising survival outcomes with a median PFS of 8.4 months and a median OS of 17.7 months. This finding indirectly suggests that HFRT combined with TMZ may exert a certain therapeutic effect in poor-prognosis GBM patients with MGMT non-hypermethylation, a population that usually has a poor response to standard TMZ-based chemoradiotherapy. However, due to the small sample size of this exploratory study, we were unable to conduct stratified survival analysis based on MGMT methylation status to further clarify its predictive value for the efficacy of HFRT combined with TMZ, nor could we explore the potential synergistic effect between MGMT methylation status and different BED values of HFRT. This is an important research gap in the present study, and subsequent prospective studies need to include MGMT methylation status as a key stratification factor to further optimize the individualized application of HFRT in GBM patients.

This study has several limitations. First, given the inherent limitation of the study design, including its retrospective nature, small sample size, and heterogeneous patient population, the preliminary findings warrant further validation in well-designed, large-sample prospective clinical trials. Second, the lack of molecular marker data precluded an assessment of their impact on HFRT efficacy, and the absence of quality-of-life evaluations limited comprehensive toxicity profiling. Third, the dose threshold was not uniform. Prospective evidence-based data remain lacking for the RPA class IV–VI poor-prognosis GBM population. The NCCN guideline recommendation of a per-fraction dose ≥3 Gy for HFRT is mainly derived from clinical data in elderly GBM patients or those with poor performance status, and the optimal dose threshold for RPA class IV–VI GBM patients has not been validated by randomized controlled trials. In addition, individualized dose adjustment lacked a unified quantitative scoring system and was performed only empirically based on routine clinical and imaging parameters, introducing a certain degree of subjectivity into dose selection. The present study did not investigate the dose-effect relationship of HFRT; we were unable to clarify differences in clinical efficacy and treatment-related toxicity across different per-fraction doses (3 Gy, 3.5 Gy, 4.5 Gy) or different BED values (70 Gy, 80 Gy, 90 Gy), thus failing to identify the optimal dose cutoff for poor-prognosis GBM patients. Fourth, owing to the small sample size, the study could not perform stratified survival analyses according to MGMT methylation status, which hinders further elucidation of the predictive value of MGMT methylation for the efficacy of HFRT plus TMZ.

Future prospective studies should focus on establishing a quantitative and standardized system for determining dose thresholds by integrating clinical characteristics, advanced imaging techniques (e.g., DTI, amino acid PET), and molecular biomarkers (e.g., MGMT methylation status), as well as conducting dose-escalation studies to define the optimal HFRT dose regimen for poor-prognosis GBM patients.

## Conclusion

In this exploratory single-center retrospective study, individually tailored HFRT combined with concurrent TMZ demonstrated promising preliminary survival outcomes and well-controllable acute treatment-related toxicity in poor-prognosis GBM patients who were unsuitable for standard radiotherapy regimens. The high rates of marginal and out-of-field recurrence observed in this small sample cohort suggest a potential need for optimized target volume delineation strategies for HFRT in this specific population, such as integrating advanced imaging techniques to refine CTV boundaries. HFRT represents a potential viable therapeutic alternative for this challenging poor-prognosis GBM population, and the preliminary findings of this study provide a rational basis for hypothesis generation and the design of subsequent large-sample, multicenter prospective validation studies. These future studies are needed to further confirm the clinical efficacy of HFRT and optimize the HFRT dose regimen and target volume delineation strategies for this specific patient group.

## Data Availability

The raw data supporting the conclusions of this article will be made available by the authors, without undue reservation.

## Glossary

GBM: Glioblastoma
RPA: Recursive Partitioning Analysis
HFRT: Hypofractionated Radiotherapy
TMZ: Temozolomide
IDH: Isocitrate Dehydrogenase
WHO: World Health Organization
EORTC: European Organization for Research and Treatment of Cancer
RTOG: Radiation Therapy Oncology Group
KPS: Karnofsky Performance Status
PTV: Planning Target Volume
CTV: Clinical Target Volume
BED: Biologically Effective Dose
SIB: Simultaneous Integrated Boost
PFS: Progression-Free Survival
OS: Overall Survival
ORR: Objective Response Rate
DCR: Disease Control Rate
CR: Complete Response
PR: Partial Response
SD: Stable Disease
PD: Progressive Disease
RANO: Response Assessment in Neuro-Oncology
MRI: Magnetic Resonance Imaging
CT: Computed Tomography
VMAT: Volumetric Modulated Arc Therapy
ICRU: International Commission on Radiation Units and Measurements
DWI: Diffusion-Weighted Imaging
DSC: Dynamic Susceptibility Contrast
MRS: Magnetic Resonance Spectroscopy
MGMT: O⁶-Methylguanine-DNA Methyltransferase
MMSE: Mini-Mental Examination
rhG-CSF: Recombinant Human Granulocyte Colony-Stimulating Factor
DTI: Diffusion Tensor Imaging
¹⁸F-FET: O-(2-[¹⁸F]Fluoroethyl)-L-Tyrosine
PET: Positron Emission Tomography
TTF: Tumor-Treating Fields
OARs: Organs-at-Risk.
